# Health-Related Behaviors Among School-Aged Children and Adolescents During the Spanish Covid-19 Confinement

**DOI:** 10.3389/fped.2020.00573

**Published:** 2020-09-11

**Authors:** Rubén López-Bueno, Guillermo F. López-Sánchez, José A. Casajús, Joaquín Calatayud, Alejandro Gil-Salmerón, Igor Grabovac, Mark A. Tully, Lee Smith

**Affiliations:** ^1^Department of Physical Medicine and Nursing, University of Zaragoza, Zaragoza, Spain; ^2^Faculty of Sport Sciences, University of Murcia, Murcia, Spain; ^3^Faculty of Health Sciences, University of Zaragoza, Zaragoza, Spain; ^4^Exercise Intervention for Health Research Group (EXINH-RG), Department of Physiotherapy, University of Valencia, Valencia, Spain; ^5^Polibienestar Research Institute, University of Valencia, Valencia, Spain; ^6^Department of Social and Preventive Medicine, Centre for Public Health, Medical University of Vienna, Vienna, Austria; ^7^Institute of Mental Health Sciences, School of Health Sciences, Ulster University, Newtownabbey, United Kingdom; ^8^Cambridge Centre for Sport and Exercise Science, Anglia Ruskin University, Cambridge, United Kingdom

**Keywords:** children, adolescents, health-related behaviors, lifestyle habits, coronavirus disease, confinement

## Abstract

In response to the coronavirus disease 2019 (Covid-19) world pandemic, affected countries such as Spain enacted measures comprising compulsory confinement as well as restrictions regarding free movement. Such measures likely influence children's and adolescents' lifestyles. Our study aimed to investigate the impact that the Covid-19 confinement has on health-related behaviors (HRBs) among Spanish children and adolescents. An online survey was administered to 516 parents to collect data about 860 children and adolescents (49.2% girls) aged between 3 and 16 years in relation to physical activity, screen exposure, sleep time, and fruit and vegetable consumption during the Covid-19 confinement. Respectively, *t*-paired test and *t*-test between groups served to check differences between HRBs levels before and during the confinement as well as between strict and relaxed confinement. Significant differences were found for a reduction of weekly minutes of physical activity during the confinement (−102.5, *SD* 159.6) (*p* < 0.001), an increase of daily hours of screen exposure (2.9, *SD* 2.1) (*p* < 0.001), and a reduction of daily fruit and vegetable consumption (−0.2, *SD* 1.6) (*p* < 0.001). Sleep time showed a significant difference between strict and relaxed confinement (−0.3, *SD* 0.1) (*p* < 0.05), whereas binomial logistic regression adjusted for covariates (age, sex, education of the parents, siblings, current condition, exposure to Covid-19, and previous health risk behavior) showed significantly lower odds for screen exposure risk behavior with relaxed confinement (OR 0.60, 95%CI 0.40–0.91). The present study suggests that Covid-19 confinement reduced physical activity levels, increased both screen exposure and sleep time, and reduced fruit and vegetable consumption. Therefore, most HRBs worsened among this sample of Spanish children and adolescents. Closure of schools, online education, and the lack of policies addressing the conciliation between labor and family life could have played an important role in HRBs worsening among pupils, which might be mitigated with adequate conciliation policies, parental guidance, and community support.

## Introduction

The global pandemic of coronavirus disease 2019 (Covid-19) has forced many countries to enact confinement measures to reduce the spread of the virus (SARS-CoV-2). The Spanish Government declared a state of alarm followed by a compulsory set of measures including strict free movement restrictions implemented from 15 March 2020 onwards (1, 2). To date, these measures have been observed to be effective since new contagious have been substantially reduced, which has permitted to start with a de-escalation phase toward usual daily routine. However, the experience of a long period of confinement may have had a significant impact on those who have suffered the strictest restrictions of free movement and other potential consequences such as the problems for families derived from the lack of conciliation between labor and family life.

In this regard, the compulsory movement restriction meant the prohibition of movement of children outside households up to 6 or more weeks in a row, with no certainty about potentially damaging consequences on their health and well-being. Additionally, because 94.1% of the Covid-19-infected children do not present symptoms or have mild-to-moderate disease (3), affecting mainly the elderly with whom the minors share households with (4), social distancing measures were put in place across Europe to reduce the human-to-human infection; such measures included the closing of schools and high schools. Consequently, several pupils have been affected by the temporary closing of their schools and high schools and the adoption of online learning platforms instead (5).

The United Nations stated that the mitigation measures may inadvertently do more harm than good (6), and in this context, the closing of schools and high schools supposedly isolates and socially deprives many children of all ages around the world. Numerous studies have previously linked isolation to different conditions such as cardiovascular disease among children (i.e., elevated total cholesterol, elevated blood pressure, overweight, low high-density lipoprotein level, low maximum oxygen consumption, and elevated glycated hemoglobin concentration), and social deprivation has been observed to have a negative effect over social cognition and both emotional and motor developments (7–10). Therefore, since a substantial amount of children and adolescents might have been temporarily deprived of parental care, adequate healthy and sustained routines, and cognitive and physical stimuli for their age, research relating to this topic is urgently required. For instance, due to the Covid-19 movement restrictions, a higher homestay would be expected, which, in turn, might increase screen exposure (11); at the same time, higher levels of screen exposure might also lead to lower levels of physical activity and, eventually, lower sleep time (12, 13), which along with circadian deregulation may influence Covid-19 infection and severity (14). Furthermore, recent studies have observed a significant reduction in physical activity levels of adults during the Covid-19 confinement (15, 16). Moreover, such studies have found that adults experiencing higher reductions in physical activity levels or performing lower levels of physical activity during the Covid-19 pandemic have poor mental health and well-being (17, 18). Indeed, similar associations observing poorer mental health as a consequence of a reduction of physical activity levels due to the Covid-19 confinement might also exist for children and adolescents.

In this unprecedented situation due to Covid-19 confinement, where infection mitigation measures may have had an impact on the usual lifestyle, there are no studies yet examining how the experience of Covid-19 confinement has influenced health-related behaviors (HRBs) in children and adolescents. Therefore, the present study aimed to analyze the influence of confinement over HRBs in Spanish children and adolescents, which could contribute to informing future public health strategies as aimed at this specific population. Based on previous literature, we hypothesized that both strict and relaxed Covid-19 confinement would be associated with unfavorable HRBs when compared with pre-Covid-19 confinement setting. Indeed, stronger associations between strict confinement and worse HRBs are expected.

## Methods

A parent-reported questionnaire was conducted to assess associations between phases of confinement and HRBs during the Covid-19 pandemic in children and adolescents.

### The Survey

A web-form link served to collect data regarding HRBs during the period 22 March to 10 May 2020 (i.e., from the 7th day of enacted national confinement in Spain up to the 15th day of relaxed confinement for children). The survey was launched on social media on 22 March 2020, together with initial information about the aim of the study. Adults residing in Spain, aged 18 years and over, having children, and currently confined due to Covid-19 were eligible to participate. Convenience sampling was used to select the participants of the study; according to server analytics, 650 adult media users covering all the Spanish regions were invited to participate. Participants were provided with an information sheet about the study aims as well as the instructions for the survey, gave informed consent to participate, and confirmed the confined status of their children. Provided data were anonymously treated in accordance with Spanish law for general data protection. At the end of the survey, participants were provided with recommendations regarding health habits. Overall, 516 parents provided data about 860 children and adolescents in relation to the following variables: age, gender, education of the parents, previous condition, number of siblings, phase of confinement, exposure to Covid-19, physical activity, screen exposure, sleep time, and fruits and vegetable consumption.

### Ethics

The study was conducted following the principles of the World Medical Declaration of Helsinki and was approved by the Ethics Committee of Research in Humans of the University of Valencia (register code 1278789). The study was reported accordingly to the Strengthening the Reporting of Observational Studies in Epidemiology statement (19).

### The Phase of Confinement (Exposure)

**Figure 1:**
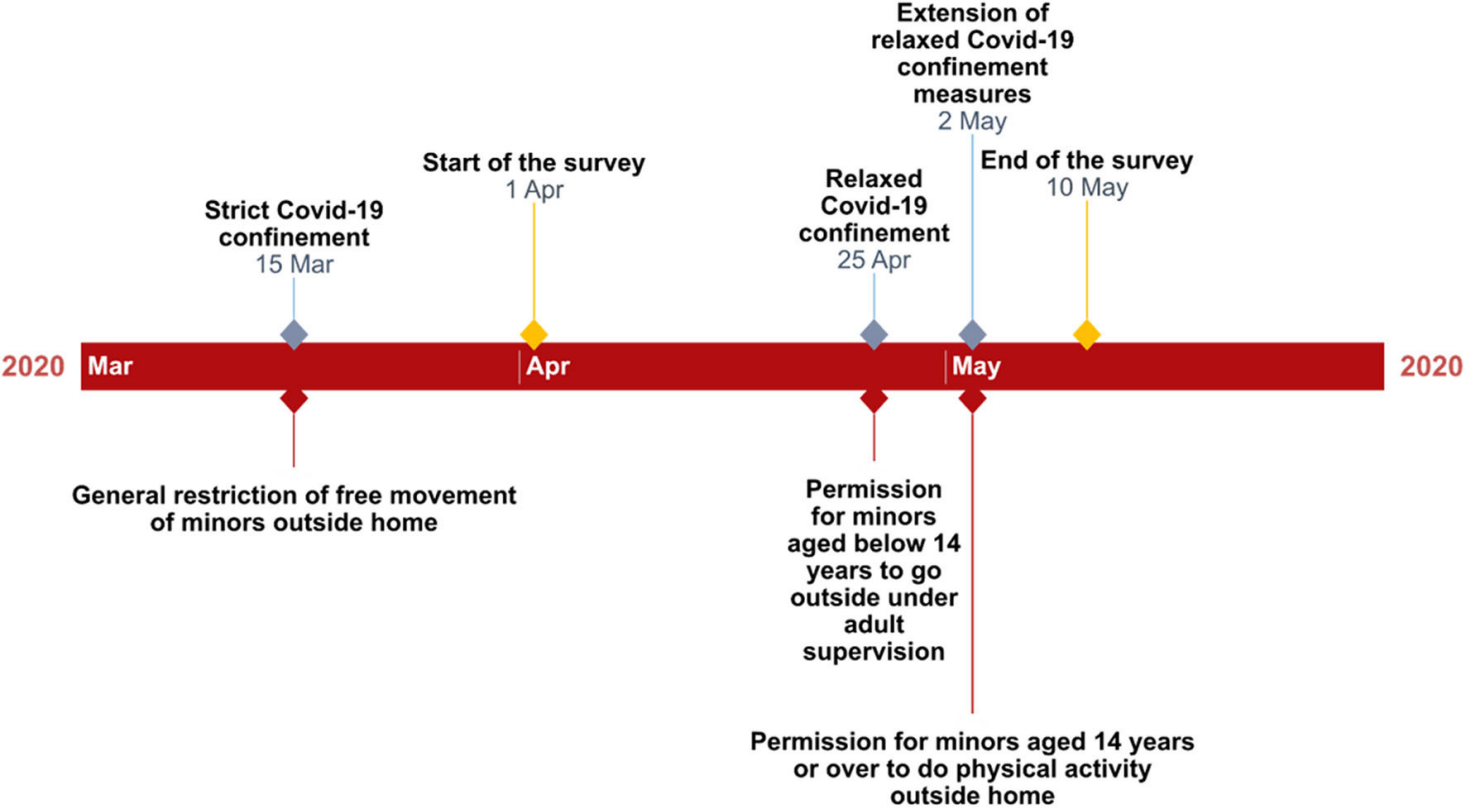
Timeline of the most important Covid-19 confinement measures regarding minors in Spain.

Data from web server corresponding to the date of completing the survey served to categorize this variable into those experiencing strict confinement (i.e., those completing the questionnaire from 1 April up to 25 April) and those experiencing relaxed confinement (i.e., those completing the questionnaire from 26 April up to 10 May). These date intervals were set in accordance to the measures enacted by the Spanish Government in relation to the Covid-19 pandemic (1, 2); strict confinement did not allow any free movement of minors outside the household if not for medical reasons or, in the case of those aged 15 or over, to do the shopping or take a dog for a short walk once a day, and, in any case, keeping a compulsory social distance of 1.5 m from others. On the other hand, relaxed confinement permitted minors aged below 14 years to go outside once a day for no more than an hour, accompanied by an adult, in a time band from 9:00 a.m. to 9:00 p.m. and keeping a social distance of 2 m from others. Also, minors aged 14 years joined the group of those aged 15 years or over to be allowed to do the same from that moment. Finally, from 2 May, those aged 14 years or over were permitted to do physical activity outside the household in two specific time bands (6:00 a.m. to 10:00 a.m. and 8:00 p.m. to 11:00 p.m.), whereas the time band for those aged below 14 years was restricted to 12:00 a.m. to 7:00 p.m. for activities outside the home. Figure 1 displays an illustration of the key dates regarding this study.

### Health-Related Behaviors (Outcome)

Outcome variables were estimated through a set of four questions included in the survey in relation to four HRBs (i.e., physical activity, screen exposure, sleep time, and fruit and vegetable consumption). The answers of the parents to the following single-item questions were used as proxy measures of their children's HRBs: “How many minutes of physical activity does your child usually perform weekly?” and answers ranging from “0” to “More than 720;” “How many hours is your child usually exposed to screens such as TV, cell phone, and tablet daily?,” with possible answers ranging from “0 h” to “more than 10 h;” “How many hours does your child usually sleep daily?, with answers comprising from “ <5 h” to “more than 10 h;” “How many pieces of fruit and vegetables do your child usually eat daily?, and possible answers ranging from “0” to “more than 5.” These questions were asked twice to the parents; first, referred to before the confinement setting and, second, regarding the confinement setting. Single-item questions used to estimate HRBs such as physical activity have shown high reproducibility [Spearman's rank correlation coefficients (*r* = 0.72–0.82)], as well as a strong agreement when meeting physical activity guidelines (kappa = 0.63, 95%CI 0.54–0.72) (20). Also, the use of parents' self-reporting to estimate children's physical activity and sedentary time has shown significant positive associations when both were accelerometer-measured (*p* < 0.001) (21).

Based on current guidelines and relevant research, a corresponding health risk behavior was defined for each HRB as follows: <420 weekly minutes of physical activity (physical activity); more than 2 h of daily screen time (screen exposure); <9 daily sleep hours (sleep time); and <3 pieces of fresh fruits and vegetables a day (fruit and vegetable consumption) (22–25). For each HRB, participants were categorized into those meeting the definition of health risk behavior and those not meeting the definition of health risk behavior.

### Covariates

According to previous research (26–28), the present study also estimated age, gender, education of the parents, previous condition, number of siblings, and exposure to Covid-19. Responses of the parents regarding their children were categorized as follows: age [cutoff points for years were set according to current school stages in Spain: infants (3–5 years), primary (6–12 years), and secondary (13–16 years)]; education of the parents (“any of the parents holding a university degree” or “none of the parents holding a university degree”); current condition (“experiencing any physical or mental condition” or “not experiencing any physical or mental condition;” number of siblings (“having one or more siblings” or “not having any sibling”); and Covid-19 exposure (“infected with Covid-19 or close to an infected person” or “not exposed”). Finally, the previous health risk behavior of the children (i.e., meeting or not meeting the definition of health risk behavior before the Covid-19 confinement) was also considered.

### Statistical Analyses

Statistical analyses were conducted through Stata version 16.1 (StataCorp, Texas, USA). The Kołmogorov–Smirnov test served to check normality. Differences between each HRB before and during the confinement as well as between strict and relaxed confinement were respectively assessed using paired *t*-test and *t*-test between groups. Also, the effect size for these differences was checked calculating Cohen's d. Associations between type of confinement due to Covid-19 and each HRB were assessed using binomial logistic regressions adjusted for covariates, providing adjusted odds ratios (ORs) and 95% CIs for the whole sample. We also conducted stratified analyses to check associations concerning gender and age. Finally, propensity score with nearest-neighbor matching served to further check the differences between strict and relaxed confinement for each HRB. Participants with missing data in any study variable were discarded for the study (*n* = 45). Levels of significance were set at *p* < 0.05.

## Results

The characteristics of the sample are presented in Table 1. A total of 860 children and adolescents on average aged 9.6 (*SD* 3.9) participated in this study. Of those, 423 (49.2%) were girls, and 611 (71.1%) were experiencing strict confinement. At the time of the questionnaire reply, 35 (4.1%) participants declared having being exposed to Covid-19 and 144 (16.7%) having a previous condition. Overall, the number of participants with siblings is 692 (80.5%), whereas that with any parent holding a university degree represents 28.8% of the sample.

**Table 1:** Characteristics of the study sample.

***N* = 860**	***n* (%)**	**Mean (*SD*)**
**Gender**		
Boys	437 (50.8)	
Girls	423 (49.2)	
**Age**		9.6 (3.9)
**Confinement**		
Strict	611 (71.1)	
Relaxed	249 (28.9)	
**Education of the Parents**		
Holding a university degree	248 (28.8)	
Not holding a university degree	612 (71.2)	
**Siblings**		
Yes	692 (80.5)	
No	168 (19.5)	
**Current Condition**		
Yes	144 (16.7)	
No	716 (83.3)	
**Exposure to Covid-19**		
Yes	35 (4.1)	
No	825 (95.9)	
**Physical Activity (weekly minutes)**		
Before confinement		198.6 (180.9)
During strict confinement		95.5 (123.8)
During relaxed confinement		97.8 (121.4)
**Screen Exposure (h/day)**		
Before confinement		2.0 (1.6)
During strict confinement		4.9 (2.3)
During relaxed confinement		4.8 (2.3)
**Sleep Time (h/day)**		
Before confinement		9.1 (1.2)
During strict confinement		9.3 (1.6)
During relaxed confinement		9.0 (1.7)
**Fruit and Vegetable**		
**Consumption (portion/day)**		
Before confinement		3.2 (2.0)
During strict confinement		3.1 (2.1)
During relaxed confinement		2.8 (1.9)

Table 2 shows previous levels for each HRB as well as differences between before and during Covid-19 confinement. Previous physical activity levels were estimated in 198.6 (*SD* 180.9) weekly minutes for the whole sample; boys and younger participants reached the highest levels with 211.9 (*SD* 188.4) and 223.0 (*SD* 198.0) weekly minutes, respectively. Screen exposure was overall estimated as 2.0 (*SD* 1.6) daily hours; boys 2.1 (*SD* 1.7) and older participants reach the highest levels 2.4 (*SD* 1.3) for this HRB. Concerning daily sleep hours, these are estimated in 9.1 (*SD* 1.2) for the entire sample; the subgroup of younger participants shows the highest values for this HRB: 9.8 (*SD* 1.2). Finally, daily fruit and vegetable consumption is estimated in 3.2 (*SD* 2.0) pieces for all the participants: boys with 3.3 (*SD* 2.1), and those from the younger subgroup present the highest values for this HRB with 3.7 (*SD* 2.1) daily pieces in subgroup comparisons. Overall physical activity and all gender and age subgroups display significant reduction between before and during the confinement; the subgroup of participants aged between 6 and 12 years displays the highest reduction of weekly minutes [−120.4 (*SD* 159.0)]. Also, screen exposure shows a significant increase for all the participants as well as for all subgroups; the highest increase is observed for older participants who show 3.3 (*SD* 2.1) more daily hours. Sleep time presents significant differences only for the younger and older participant subgroups with, respectively −0.4 (*SD* 1.8) and 0.6 (*SD* 1.7) daily hours. Concerning daily fruits and vegetable consumption, all the subgroups present significant reductions with the exception of the subgroup of older participants; the highest reductions are presented in the subgroup of the younger participants [−0.6 (*SD* 2.0)].

**Table 2:** Differences regarding health-related behaviors before and during Covid-19 confinement.

	***n* (%)**	**Before confinement Mean (*SD*)**	**During confinement Mean (*SD*)**	**Difference (before and during confinement) (*SD*)**	***t***	**df**	***p-value^a^***	***d^b^***
**Physical Activity**								
**(min/week)**								
**Overall (*****n*** **=** **860)**		198.6 (180.9)	96.1 (123.0)	−102.5 (159.6)	18.8	859	0.0000	0.66
**Gender**								
Boys	437 (50.8)	211.9 (188.4)	104.8 (130.0)	−107.1 (170.2)	13.1	436	0.0000	0.66
Girls	423 (49.2)	184.9 (172.0)	87.2 (114.9)	−97.7 (148.0)	13.6	422	0.0000	0.67
**Age**								
≥3 and ≤5 years	162 (18.8)	223.0 (198.0)	131.0 (160.2)	−92.0 (174.0)	6.7	161	0.0000	0.51
≥6 and ≤12 years	459 (53.4)	209.0 (180.0)	88.6 (112.4)	−120.4 (159.0)	16.2	458	0.0000	0.80
≥13 and ≤16 years	239 (27.8)	162.1 (165.3)	86.9 (109.3)	−75.2 (146.4)	7.9	238	0.0000	0.54
**Screen Exposure (h/day)**								
**Overall (*****n*** **=** **860)**		2.0 (1.6)	4.9 (2.3)	2.9 (2.1)	39.4	859	0.0000	1.43
**Gender**								
Boys	437 (50.8)	2.1 (1.7)	5.0 (2.4)	2.9 (2.2)	28.0	436	0.0000	1.43
Girls	423 (49.2)	1.9 (1.5)	4.7 (2.3)	2.8 (2.1)	27.7	422	0.0000	1.44
**Age**								
≥3 and ≤5 years	162 (18.8)	1.7 (1.9)	3.9 (2.0)	2.2 (2.4)	11.6	161	0.0000	1.13
≥6 and ≤12 years	459 (53.4)	1.9 (1.5)	4.7 (2.3)	2.9 (2.0)	30.8	458	0.0000	1.46
≥13 and ≤16 years	239 (27.8)	2.4 (1.3)	5.8 (2.3)	3.3 (2.1)	24.7	238	0.0000	1.74
**Sleep Time (h/day)**								
**Overall (*****n*** **=** **860)**		9.1 (1.2)	9.2 (1.6)	0.1 (1.8)	1.5	859	0.1288	0.06
**Gender**								
Boys	437 (50.8)	9.1 (1.2)	9.3 (1.6)	0.2 (1.7)	1.9	436	0.0648	0.11
Girls	423 (49.2)	9.1 (1.2)	9.1 (1.7)	0.0 (1.8)	0.3	422	0.7635	0.02
**Age**								
≥3 and ≤5 years	162 (18.8)	9.8 (1.2)	9.4 (1.7)	−0.4 (1.8)	3.1	161	0.0025	0.30
≥6 and ≤12 years	459 (53.4)	9.2 (1.1)	9.2 (1.6)	0.0 (1.7)	0.1	458	0.9563	0.00
≥13 and ≤16 years	239 (27.8)	8.5 (1.0)	9.1 (1.7)	0.6 (1.7)	5.4	238	0.0000	0.44
**Fruit and Vegetable**								
**Consumption (portion/day)**								
**Overall (*****n*** **=** **860)**		3.2 (2.0)	3.0 (2.1)	−0.2 (1.6)	3.4	859	0.0007	0.09
**Gender**								
Boys	437 (50.8)	3.3 (2.1)	3.1 (2.2)	−0.2 (1.6)	2.3	436	0.0213	0.08
Girls	423 (49.2)	3.1 (1.9)	2.9 (1.9)	−0.2 (1.6)	2.5	422	0.0125	0.11
**Age**								
≥3 and ≤5 years	162 (18.8)	3.7 (2.1)	3.1 (2.1)	−0.6 (2.0)	3.7	161	0.0003	0.28
≥6 and ≤12 years	459 (53.4)	3.1 (2.0)	2.9 (2.0)	−0.2 (1.5)	2.7	458	0.0064	0.10
≥13 and ≤16 years	239 (27.8)	3.0 (2.0)	3.1 (2.1)	0.1 (1.4)	1.1	238	0.2800	0.05

a *t-paired test (before and during confinement)*.

b *Cohen's d: small 0.20; medium 0.50; large 0.80*.

Table 3 shows differences concerning HRBs between strict and relaxed Covid-19 confinement. Sleep time is the only HRB that shows significant differences in both overall and specific subgroups such as boys [−0.4 (*SD* 0.2) daily sleep hours] and participants aged between 6 and 12 years [−0.3 (*SD* 0.2) daily sleep hours].

**Table 3:** Differences regarding health-related behaviors between strict and relaxed Covid-19 confinement.

	***n* (%)**	**Strict confinement Mean (*SD*)**	**Relaxed confinement Mean (*SD*)**	**Difference (strict and relaxed confinement) (*SD*)**	***t***	**df**	***p*-value*^a^***	***d^b^***
**Physical Activity**								
**(minutes/week)**								
**Overall (*****n*** **=** **860)**		95.5 (123.8)	97.8 (121.4)	2.3 (9.3)	0.2	858	0.8051	0.02
**Gender**								
Boys	437 (50.8)	103.8 (130.0)	107.4 (130.6)	3.6 (13.7)	0.2	435	0.7924	0.03
Girls	423 (49.2)	86.9 (116.7)	87.9 (110.8)	1.0 (12.3)	0.1	421	0.9341	0.01
**Age**								
≥3 and ≤5 years	162 (18.8)	119.8 (148.0)	156.2 (183.7)	36.4 (27.2)	1.3	160	0.1826	0.23
≥6 and ≤12 years	459 (53.4)	90.2 (119.9)	84.9 (93.2)	−5.3 (11.4)	0.5	457	0.6414	0.05
≥13 and ≤16 years	239 (27.8)	89.6 (113.0)	78.8 (99.5)	−10.8 (16.3)	0.7	237	0.5098	0.10
**Screen Exposure (h/day)**								
**Overall**		4.9 (2.3)	4.8 (2.3)	0.1 (0.2)	0.8	858	0.4345	0.06
**Gender**								
Boys	437 (50.8)	5.1 (2.4)	4.8 (2.3)	−0.3 (0.2)	1.2	435	0.2075	0.13
Girls	423 (49.2)	4.7 (2.3)	4.7 (2.4)	0.0 (0.2)	0.2	421	0.8478	0.02
**Age**								
≥3 and ≤5 years	162 (18.8)	4.0 (1.9)	3.9 (2.2)	−0.1 (0.3)	0.3	160	0.7290	0.06
≥6 and ≤12 years	459 (53.4)	4.7 (2.3)	4.8 (2.3)	0.1 (0.2)	0.4	457	0.6543	0.05
≥13 and ≤16 years	239 (27.8)	5.9 (2.3)	5.4 (2.4)	−0.4 (0.3)	1.2	237	0.2315	0.18
**Sleep Time (h/day)**								
**Overall**		9.3 (1.6)	9.0 (1.7)	**−0.3 (0.1)**	2.3	858	0.0209	0.17
**Gender**								
Boys	437 (50.8)	9.4 (1.6)	9.0 (1.6)	**−0.4 (0.2)**	2.2	435	0.0255	0.23
Girls	423 (49.2)	9.2 (1.6)	9.0 (1.8)	−0.2 (0.2)	1.0	421	0.2956	0.11
**Age**								
≥3 and ≤5 years	162 (18.8)	9.5 (1.6)	9.2 (1.8)	−0.3 (0.3)	0.9	160	0.3715	0.15
≥6 and ≤12 years	459 (53.4)	9.3 (1.6)	9.0 (1.7)	**−0.3 (0.2)**	2.1	457	0.0350	0.21
≥13 and ≤16 years	239 (27.8)	9.2 (1.6)	9.0 (1.8)	−0.2 (0.2)	0.8	237	0.4010	0.13
**Fruit and Vegetable**								
**Consumption (portion/day)**								
**Overall**		3.1 (2.1)	2.8 (1.9)	−0.2 (0.2)	1.6	858	0.1127	0.12
**Gender**								
Boys	437 (50.8)	3.2 (2.3)	2.9 (1.9)	−0.3 (0.2)	1.4	435	0.1554	0.15
Girls	423 (49.2)	2.9 (1.9)	2.8 (2.0)	−0.2 (0.2)	0.8	421	0.4356	0.08
**Age**								
≥3 and ≤5 years	162 (18.8)	3.2 (2.0)	3.1 (2.4)	−0.1 (0.4)	0.2	160	0.8463	0.03
≥6 and ≤12 years	459 (53.4)	3.0 (2.1)	2.7 (1.8)	−0.3 (0.2)	1.3	457	0.2073	0.13
≥13 and ≤16 years	239 (27.8)	3.2 (2.2)	2.8 (1.9)	−0.3 (0.3)	1.1	237	0.2828	0.16

a *t-test between groups (strict and relaxed confinement)*.

b *Cohen's d: small 0.20; medium 0.50; large 0.80*.

*Bold values mean significant values (p < 0.05)*.

Adjusted ORs for each health risk behavior during COVID-19 confinement are presented in Table 4. Solely screen exposure shows significant odds reduction of health risk behavior overall (OR 0.60 95%CI 0.40–0.91) as well as in girls (OR 0.55 95%CI 0.31–0.99) and participants aged between 6 and 12 years (OR 0.26 95%CI 0.03–0.92).

**Table 4:** Adjusted odds ratios (95% confidence interval) for each health risk behavior during Covid-19 confinement in the entire study population and in age and gender subgroups (reference group: strict confinement).

***n* = 860**	**Confinement**	***n* (%)**	**Physical activity**		**Screen exposure**		**Sleep time**		**Fruits and vegetable consumption**	
			**Model 1*^a^***	**Model 2*^b^***	**Model 1*^a^***	**Model 2*^b^***	**Model 1*^a^***	**Model 2*^b^***	**Model 1*^a^***	**Model 2*^b^***
**All**	Strict	611 (71.1)	1	1	1	1	1	1	1	1
	Relaxed	249 (29.0)	0.98 (0.50–1.92)	0.72 (0.34–1.54)	**0.66 (0.44–0.99)**	**0.60 (0.40–0.91)**	1.40 (1.03–1.93)	1.31 (0.95–1.81)	1.13 (0.77–1.66)	0.79 (0.46–1.36)
**Gender**										
Boys	Strict	311 (71.2)	1	1	1	1	1	1	1	1
	Relaxed	126 (28.8)	1.04 (0.44–2.42)	0.78 (0.30–2.03)	0.66 (0.36–1.20)	0.63 (0.34–1.19)	1.34 (0.86–2.11)	1.30 (0.81–2.09)	0.93 (0.56–1.56)	0.60 (0.28–1.28)
Girls	Strict	300 (70.9)	1	1	1	1	1	1	1	1
	Relaxed	123 (29.1)	0.85 (0.28–2.60)	0.74 (0.20–2.71)	0.66 (0.38–1.14)	**0.55 (0.31–0.99)**	1.47 (0.95–2.28)	1.31 (0.83–2.05)	1.42 (0.80–2.54)	1.03 (0.47–2.28)
**Age**										
≥3 and ≤5 years	Strict	112 (69.1)	1	1	1	1	1	1	1	1
	Relaxed	50 (30.9)	0.51 (0.19–1.39)	0.36 (0.11–1.22)	0.60 (0.29–1.27)	0.57 (0.26–1.23)	1.17 (0.55–2.49)	1.02 (0.46–2.62)	0.97 (0.43–2.17)	1.10 (0.28–4.29)
≥6 and ≤12 years	Strict	320 (69.7)	1	1	1	1	1	1	1	1
	Relaxed	139 (30.3)	2.10 (0.57–7.17)	1.10 (0.26–4.39)	0.81 (0.48–1.38)	0.78 (0.45–1.36)	1.38 (0.90–2.11)	1.25 (0.80–1.93)	1.36 (0.79–2.32)	0.90 (0.44–1.84)
≥13 and ≤16 years	Strict	179 (74.9)	1	1	1	1	1	1	1	1
	Relaxed	60 (25.1)	1.05 (0.20–5.45)	0.66 (0.11–4.16)	**0.31 (0.09–0.99)**	**0.26 (0.03–0.92)**	1.62 (0.88–2.96)	1.60 (0.86–2.98)	0.92 (0.44–1.94)	0.31 (0.09–1.05)

a *Adjusted for age and gender (all participants), for gender (age categories), and for age (boys, girls)*.

b *Model 1 + education, siblings, current condition, exposure to Covid-19, and previous health risk behavior*.

*Bold values mean significant values (p < 0.05)*.

Finally, Table 5 shows the average treatment effect on treatment (relaxed confinement participants), in which none of the HRBs present significant treatment effects.

**Table 5:** Differences between relaxed confinement and strict confinement for each health-related behavior.

**Health-related behavior**	**ATT**			
	**Treated**	**Control**	**Difference (*SD*)**	***p***
Physical activity (min/week)	97.8	90.0	7.8 (9.5)	0.393
Screen exposure (h/day)	4.8	4.9	−0.1 (0.2)	0.464
Sleep time (h/day)	9.0	9.1	−0.1 (0.2)	0.270
Fruits and vegetable consumption (portion/day)	2.8	3.1	−0.3 (0.2)	0.105

*ATT, average treatment effect on treated calculated as a difference for each health-related behavior between the relaxed confinement (treated) and strict confinement (control); difference, treated—controls; SD, standard deviation*.

## Discussion

The present study provides novel data from an unprecedented set of public health measures restricting the mobility of children and adolescents as a result of the Covid-19 pandemic. The most relevant finding in this study with an important sample of the Spanish child and adolescent population was that overall examined HRBs, except for sleep time, worsened with the confinement. Also, only levels and odds of health risk behavior for screen exposure significantly improved during the 1st weeks of relaxed confinement, although further analyses did not confirm such a trend. Values for physical activity, screen exposure, and fruit and vegetable consumption also worsened during the confinement in all gender and age subgroups except for ≥13 and ≤16 years' subgroup for the last case (i.e., fewer minutes of weekly physical activity, more hours of daily screen exposure, and less daily pieces of fruit and vegetable consumed), whereas only screen exposure improved (i.e., less time exposed to screens).

### Physical Activity

The association between social isolation and lower levels of physical activity in children was reported a few decades ago (29). More recently, a reduction of physical activity (i.e., 2.30 h/week) has been found in prior research regarding confined children and adolescents due to Covid-19 (30); such values were higher than those found in the present study, which observed a difference of 1.40 h/week in respect to previous confinement levels. Differences between the two studies concerning physical activity could be attributed to different sample characteristics; for instance, children from higher socioeconomic backgrounds have shown higher levels of physical activity, whereas the context of confinement might influence physical activity levels (e.g., more time confined or experiencing stricter confinement can modify ordinary levels of physical activity) (28–32). Closure of schools during both strict and relaxed confinement might play a key role in this found reduction since schools, and, particularly physical education classes provide an adequate environment to promote active behaviors among children and adolescents (33–35). Also, current research does not show evidence enough to consider the possibility that such activity behaviors could be compensated at home (36, 37). Finally, because healthy habits such as active commuting to schools (i.e., walking or cycling), which has been associated with increasing overall physical activity, and reducing sedentary behaviors (38, 39), have been restricted during both strict and relaxed confinement, it was difficult to expect that those physically active behaviors could be compensated at home; this might partially explain the finding of a recent study observing adolescents living in rural areas as more prone to reduce their physical activity levels during the Covid-19 pandemic when compared with their rural counterparts (40). Particularly, adolescents with lower physical fitness were observed to greatly reduce their physical activity levels during the Covid-19 pandemic (40, 41).

### Screen Exposure

With higher time spent at home, it was expected that screen exposure could reach higher levels than before the Covid-19 confinement. A recent study found screen exposure to increase by 4 h/day among Italian children and adolescents during Covid-19 strict confinement (30). Furthermore, online gaming and streaming activity have been observed to rise in different countries during the Covid-19 confinement (42). Also, ordinary school attendance has been largely substituted by both digital homework and digital classes in Spain, which could have added more screen time to the already existing before the confinement. Particularly, mobile phones could have had a significant impact on teenagers' socialization processes and have added more screen time while confined (43); the lower odds for health risk behavior observed for the ≥13 and ≤16 years' subgroups with the relaxed confinement could be also explained by higher opportunities to physically meet their friends. Moreover, since Spanish workers have been instructed to telework at home during the Covid-19 confinement, the use of electronic devices might have contributed to increasing children's behavior as regards exposure to screen since there is a possibility of children being influenced by their parent's behaviors regarding this issue; family environment concerning screen exposure has been observed to directly associate with children's exposure to screens (44). Further, the increase of hours exposed in front of a screen observed in this study might also explain the lower levels of physical activity found since higher screen time has been previously associated with lower physical activity among Spanish adolescents (12).

### Sleep Time

Because children were experiencing a change as regards their usual daily habits, it was expected to find different sleep times in this study; overall, sleep time variations are normal among children aged between 3 and 7 years (45). However, contrary to expected, sleep time tended to slightly increase during the confinement, even though higher screen time and lower physical activity could have reduced sleep time since the opposite was observed in prior research with children (46, 47). However, this increase was similar to what was found in another study among Italian children and adolescents during the Covid-19 confinement (30); thus, this situation might have contributed to reinforcing timetables, which, in turn, might have been useful to mitigate potential detrimental effects over adequate sleep time (48). In contrast, a recent study among French adults found that 47% reported a decrease in sleep quality during quarantine, with sleep reduction being the most associated factor (49).

### Fruit and Vegetable Consumption

There is no prior evidence on how Covid-19 confinement might have influenced eating habits as regards fruits and vegetables among children and adolescents. Interestingly, our study found an important significant reduction of fruit and vegetable consumption during the Covid-19 confinement for the subgroup of children aged between 3 and 5 years. A possible explanation for this finding might be related to difficulties balancing family and working life since many parents have had to telework while taking care of their children during the Covid-19 pandemic; this situation might have led to poorer eating habits, particularly among those children potentially less independent (i.e., younger children). Similarly, a study with Italian children and adolescents confined due to Covid-19 pandemic showed higher red meat, potato chip, and sugary drink consumption (30), which suggests that, as observed in the present study, eating habits overall might have worsened during the confinement. In contrast, a recent survey among 600 Spanish adults reported that most of the participants maintained vegetable and fruit consumption during the first 5 confinement weeks, although these results might have changed after the whole confinement period (50). Also, lower food away from home consumption was linked to changes in diet quality (i.e., less added sugars and added fats, and more fiber consumption) (51), although, for this specific and unprecedented context differences regarding usual eating habits and socioeconomic family status (i.e., higher family socioeconomic status usually lead to healthier eating habits), self-regulating behaviors and knowledge might play an important role (52). Also, school canteen deprivation could influence the fruit and vegetable consumption since that has observed to worsen healthy habits concerning diet (53). Besides, since the likelihood of eating at convenience during Covid-19 confinement might have increased, that could have contributed to maintaining similar levels of fruit and vegetable consumption during this period as the active choice could benefit this behavior among children (54).

Overall, both parental guidance and example can strongly influence children's and adolescent behaviors regarding diet, screen, and physical activity habits (55, 56), which, in turn, could be also influenced by socioeconomic variables such as education and income (i.e., higher education or higher income may lead to healthier HRB) (52, 57). Health complications for children and adolescents derived from prolonged confinement or repeated viruses' outbreaks might comprise a higher fat percentage, lower bone mineral density, lower motor competence, higher blood pressure, and higher socio-emotional behavior problems later, among others (58–63). Therefore, further research examining longitudinal consequences of confinement over objectively assessed health behaviors (e.g., use of accelerometers for estimating physical activity) would contribute to better understand the reasons for changes in HRBs as well as to define better strategies aimed at vulnerable populations such as children and adolescents; also, the study of adaptive patterns for HRBs as those observed for adults during the Covid-19 confinement could contribute to better understand the nature and severity of these changes (28).

### Strengths and Limitations

Strengths of the current study consist of examining a large and well-disseminated sample of Spanish children and adolescents (i.e., participants from all the Spanish regions), and the analysis of an important set of control variables such as previous health conditions or exposure to Covid-19 in the referred time. Besides, the trends for specific HRBs such as physical activity or screen exposure remain consistent overall as well as in subgroup analyses. On the other hand, an important limitation of this study was self-reported data by parents, which may lead to recall bias; as suggested by Thorn et al. (64) outcomes such as physical activity, screen, and diet reported by children possibly lead to different estimations as regards their parents. Owing to their brevity, single-item questions have been recommended to apply in specific contexts of illness and frailty (65); thereby, the authors decided to use it in this specific context of confinement due to Covid-19 pandemic even though these specific questions were not specifically validated. Also, the convenience sampling method used to recruit participants might lead to a selection bias, which, in turn, could have shown a biased estimation of the study variables concerning the study population; thus, interpretation of the results of this study should be made in the light of this information. Further, the wide age range used for this study hampers generalizations on populations of children of a specific age. Last, the observational design of the study does not allow us to infer any causality.

## Conclusion

The results found in the present study suggest that Covid-19 confinement substantially reduced physical activity levels, increased both screen exposure and sleep time, and reduced fruit and vegetable consumption; thus, most of HRBs worsen among a sample of Spanish children and adolescents. Also, the 1st weeks of confinement did not seem to significantly improve HRBs, except sleep time. Restrictive mobility measures with the closure of schools and high schools could have played an important role in this HRB worsening, which could be mitigated with policies for labor and family time conciliation, parental guidance, and community support.

## Data Availability Statement

The raw data supporting the conclusions of this article will be made available by the authors, without undue reservation.

## Ethics Statement

The studies involving human participants were reviewed and approved by Ethics Committee of Research in Humans of the University of Valencia. Written informed consent to participate in this study was provided by the participants' legal guardian/next of kin.

## Author Contributions

RL-B, GL-S, AG-S, IG, LS, and JC contributed to the conception and design of the study. RL-B organized the database and wrote the first draft of the manuscript. RL-B and LS performed the statistical analysis. JC, AG-S, LS, IG, MT, and JAC wrote sections of the manuscript. All authors contributed to the article and approved the submitted version.

## Conflict of Interest

The authors declare that the research was conducted in the absence of any commercial or financial relationships that could be construed as a potential conflict of interest.
